# Resistance to Degradation of Silk Fibroin Hydrogels Exposed to Neuroinflammatory Environments

**DOI:** 10.3390/polym15112491

**Published:** 2023-05-28

**Authors:** Mahdi Yonesi, Milagros Ramos, Carmen Ramirez-Castillejo, Rocío Fernández-Serra, Fivos Panetsos, Adrián Belarra, Margarita Chevalier, Francisco J. Rojo, José Pérez-Rigueiro, Gustavo V. Guinea, Daniel González-Nieto

**Affiliations:** 1 Center for Biomedical Technology, Universidad Politécnica de Madrid, 28223 Pozuelo de Alarcón, Spain; mahdi.yonesi@ctb.upm.es (M.Y.); milagros.ramos@ctb.upm.es (M.R.); carmen.ramirez@ctb.upm.es (C.R.-C.); rocio.fernandez@ctb.upm.es (R.F.-S.); fj.rojo@upm.es (F.J.R.); jose.perez@ctb.upm.es (J.P.-R.); gustavovictor.guinea@ctb.upm.es (G.V.G.); 2 Departamento de Tecnología Fotónica y Bioingeniería, ETSI Telecomunicaciones, Universidad Politécnica de Madrid, 28040 Madrid, Spain; 3 Centro de Investigación Biomédica en Red de Bioingeniería, Biomateriales y Nanomedicina (CIBER-BBN), Instituto de Salud Carlos III, 28029 Madrid, Spain; 4 Departamento de Ciencia de Materiales, ETSI Caminos, Canales y Puertos, Universidad Politécnica de Madrid, 28040 Madrid, Spain; 5 Silk Biomed SL, Calle Navacerrada 18, Urb. Puerto Galapagar, 28260 Madrid, Spain; fivos@ucm.es; 6 Bioactive Surfaces SL, Puerto de Navacerrada 18. Galapagar, 28260 Madrid, Spain; 7 Neurocomputing and Neurorobotics Research Group, Faculty of Biology and Faculty of Optics, Universidad Complutense de Madrid, 28040 Madrid, Spain; 8 Biomaterials and Regenerative Medicine Group, Instituto de Investigación Sanitaria del Hospital Clínico San Carlos (IdISSC), Calle Prof. Martín Lagos s/n, 28040 Madrid, Spain; 9 Laboratorio Micro-CT UCM, Departamento de Radiología, Rehabilitación y Fisioterapia, Facultad de Medicina, Universidad Complutense de Madrid, 28040 Madrid, Spain; abelarra@ucm.es (A.B.); chevalier@med.ucm.es (M.C.)

**Keywords:** silk fibroin, degradation, hydrogels, brain, stroke, Alzheimer’s disease

## Abstract

Central nervous system (CNS) diseases represent an extreme burden with significant social and economic costs. A common link in most brain pathologies is the appearance of inflammatory components that can jeopardize the stability of the implanted biomaterials and the effectiveness of therapies. Different silk fibroin scaffolds have been used in applications related to CNS disorders. Although some studies have analyzed the degradability of silk fibroin in non-cerebral tissues (almost exclusively upon non-inflammatory conditions), the stability of silk hydrogel scaffolds in the inflammatory nervous system has not been studied in depth. In this study, the stability of silk fibroin hydrogels exposed to different neuroinflammatory contexts has been explored using an in vitro microglial cell culture and two in vivo pathological models of cerebral stroke and Alzheimer’s disease. This biomaterial was relatively stable and did not show signs of extensive degradation across time after implantation and during two weeks of in vivo analysis. This finding contrasted with the rapid degradation observed under the same in vivo conditions for other natural materials such as collagen. Our results support the suitability of silk fibroin hydrogels for intracerebral applications and highlight the potentiality of this vehicle for the release of molecules and cells for acute and chronic treatments in cerebral pathologies.

## 1. Introduction

Pathologies that affect the central nervous system (CNS) are one of the main causes of mortality and disability. Yet, to date, no effective treatments have been identified that can reverse the dysfunctional consequences of cerebral injury. Although classical research with neuroprotective molecules and stem cells has been ambitious and successful in preclinical trials and marginally successful in some clinical studies, these therapies have not presented significant accomplishments in clinical practice with patients.

A very promising research area of therapy to treat CNS diseases is related to the use of synthetic or natural materials as a support for the controlled release of drugs [1,2,3]. These materials can also lodge cells of different specifications and therapeutic capacities, increasing the survival of engrafted cells in the host tissue [4,5]. Any biomaterial used for these purposes must meet several requirements, such as innocuousness and inertness to avoid tissue inflammation [6]. In addition, to prevent the imposition of excessive mechanical forces that can impair natural tissue remodeling, the mechanical properties of the implanted biomaterial should be similar to the surrounding tissue [7]. This is even more critical in fragile tissues such as in the CNS, where any structural disruption might have profound consequences on functionality. In addition, biomaterials should have enough stability in the host tissue to maintain drugs/factors at therapeutic doses during the treatment window, which is different for each pathological context and depends on the specific targets and time required to modulate them to revert the tissue dysfunction [8]. While the degradability and structure of materials are usually tested in healthy environments [9,10,11], many pathological processes trigger inflammatory routes that lead to the accumulation of inflammatory cells, proteases, and other proteins with enzymatic activities that might impair the durability of pre-designed materials, especially those of natural origin, thereby shortening the treatment window. This concept may be relevant in applications where a balance between stability and prolonged therapeutic effects is required.

Silk fibroin (SF) is an inherently inert biocompatible natural material produced by various species of arthropods, mostly spiders [12] and some insects [13], that has the possibility of recombinant production [14]. SF has innate antioxidant [15,16], anti-microbial [17], and anti-inflammatory properties [18,19] with applicability in various healthcare fields such as cancer [20] or cardiovascular diseases [21]. This material can be fabricated in diverse formats, including hydrogels [22], fibers [23], films [24], and sponges [25,26], which emphasizes the versatility of SF’s applications. The ability of SF hydrogels to encapsulate cells, trackers, and therapeutic molecules, alongside the appropriate mechanical properties compatible with brain tissue [27], gives this particular format a wide range of possibilities for diagnosis and therapy in different pathologies, including CNS diseases [1,28,29,30]. In animal models, the intracerebral implantation of SF hydrogels is a safe and well-tolerated strategy, with no secondary inflammation reactions, sensorimotor impairment, or sleep–wake disturbances [31,32]. Such observations, alongside various promising preclinical and clinical pieces of research [33,34,35], make the cerebral route of injectable SF hydrogel formulations a promising strategy for cell and drug delivery [28,32,36]. However, this strategy has not been exploited at the clinical level, mostly due to the fact that it is largely unknown how stable this material is in the CNS during neuroinflammation.

Although SF is a biomaterial with acceptable stability when in contact with non-cerebral tissues [11,37], contradictory information has been reported in regard to such stability as well. For example, using contrast-enhanced ultrasound imaging, Li and colleagues found a fast rate of degradation of silk hydrogels implanted subcutaneously [10]. In the brain, adenosine-releasing SF scaffolds were partly degraded four weeks after implantation [38]. The inflammatory features that lead to neuronal death in cerebrovascular diseases or neurodegeneration are unique, creating a complex environment with several inflammatory cell participants. Among them, microglia have a main role in the surveillance of the CNS and are susceptible to polarization towards different functional phenotypes [39]. In stroke, the inflammatory microglia contribute to increasing the extension of damage after the initial injury through the release of inflammatory and neurotoxic molecules [40]. During neurodegeneration, such as in Alzheimer’s disease, microglia also become hyperactive and likely have a substantial role in the pathogenesis and progression of this disease [41]. The inflammatory microglia increase the expression and secretion of several metalloproteinases (MMPs). Under pathological conditions, abnormal activation of MMPs leads to serious structural and functional changes in the extracellular matrix (ECM); for example, the disruption of the blood–brain barrier [42].

In general, the in vivo evaluation of biomaterials degradation, including SF in the CNS, mainly remains at a qualitative level. For example, SF porous scaffolds were partly degraded 30 days after intracerebroventricular injection in the brain tissue [38]. Furthermore, our group initially reported a moderate decrease in SF hydrogel deposits in a healthy (non-pathological) brain 30 days after implantation [31]. Subsequently, Gorenkova et al. qualitatively found good stability of SF beyond 7 weeks in a rat stroke model [32]. However, precise quantification of SF degradability in the context of neuroinflammation linked with cerebrovascular and neurodegenerative diseases has not been addressed. Due to the therapeutic opportunity and implications of SF hydrogel formulations for CNS therapies in the context of drug and cell delivery and the inhospitable inflammatory CNS for many cells and ECM components, in this study, we have quantitatively examined the stability of this specific biomaterial format. For this goal, formulations of SF reconstituted with a contrast agent, or SF functionalized with a fluorescent molecule, have been developed to assess the stability of SF hydrogels exposed to inflammatory microglia (in vitro model) and inflammatory cerebral tissue using two in vivo models of cerebrovascular (stroke) and neurodegenerative (Alzheimer’s) pathologies.

## 2. Materials and Methods

### 2.1. Animals

In vivo studies of SF implantations were performed in two different models: (1) Healthy (control) or stroke (middle cerebral artery occlusion) animals with a CD-1 genetic background (Charles River Laboratories, Wilmington, MA, USA), and (2) 5× Familial Alzheimer’s Disease (FAD) transgenic mice (Alzheimer model; 5× FAD) purchased from Charles River Laboratories with a B6SJL genetic background. The 5× FAD mice express the Swedish (K670N, M671L), Florida (I716V), and London (V717I) FAD mutations along with the human presenilin 1 (PS1) gene harboring two additional FAD mutations (M146L and L286V). The expression of both transgenes was controlled by the neural Thy1 promoter to promote tissue-specific expression in the brain. Mice were bred and maintained in the animal facility of the Center for Biomedical Technology (register number: ES280790002070). The animals were housed in social contact, with free access to food and water, and in environmental enrichment in animal rooms with controlled temperature, humidity, air renovation, and a natural 12:12 cycle. Animals were checked on a daily basis by technical personnel and veterinary services. All the animal studies were made under ethical and legal regulations, and experimental procedures were authorized by the Ethical Committee of the Universidad Politécnica de Madrid and by the regional government of Madrid (authorization number PROEX 109.1/20).

### 2.2. In Vivo Pathological Models

A mouse stroke model was generated by the permanent occlusion of the middle cerebral artery (MCAO) distally with respect to the Circle of Willis. MCAO was performed on four-month-old mice having a body weight of 25–35 g. The whole procedure has been described previously [43]. Briefly, after anesthesia with isoflurane (3% for induction, 1% for maintenance), a vertical incision was made between the right eye and ear. The temporal muscle was separated, and a small craniotomy was performed on the lateral side of the skull. The middle cerebral artery (MCA) was visualized and ligated distally to the lenticulostriate branches to interrupt the cerebral blood flow in the cortical areas. Intracerebral injection of hydrogels in the striatum was performed 24 h after MCAO.

For the degenerative model, SF was implanted in six-month-old 5× FAD mice, an age of significant accumulation of β-amyloid plaques, enhanced inflammatory response, and neurodegeneration [44].

A description of the experimental groups and number of animals used in the different in vivo studies (both pathological models) is shown in Supplementary Table S1 (Supplementary Materials).

### 2.3. In Vitro Pathological Model

The microglial cell line BV2 was generously donated by Professor Cuadrado Pastor (Instituto de Investigaciones Biomédicas Alberto Sols-CSIC-UAM, Madrid, Spain). Microglial cells were cultured in the RPMI1640 medium supplemented with 100 Units/mL Penicillin/Streptomycin, 2 mM L-glutamine, and 10% selected fetal bovine serum (Hyclone, SV30160.03, San Angelo, TX, USA). Cells were maintained at 37 °C with 5% CO_2_ and 85% relative humidity. To polarize microglial cells towards inflammatory phenotypes, BV2 cells at ~80% confluence were activated with 100 ng/mL Lipopolysaccharide (LPS) as described previously [45].

Signs of inflammatory polarization were inferred from the analysis of cell growth and morphology as well as from the enhanced secretion of pro-inflammatory factors such as nitric oxide (NO) and Tumor Necrosis Factor-α (TNF-alpha). The in-circularity ratio (IC) was calculated by counting the number of amoeboid-shaped cells in random places of the dish and dividing by the total number of cells in every image field. At least three different visual fields were analyzed per group. Immediately after imaging acquisition, the cells medium was collected, centrifuged, and the supernatant was used to determine the NO and TNF-alpha content. The attached cells were washed with Phosphate-Buffered Saline (PBS), detached after trypsinization, and manually counted in a hemocytometer.

The NO determination was performed following the Griess methodology [46]. Briefly, the cell culture conditional medium was treated with the reagent of Griess, composed of sulfanilamide (Sigma Aldrich, S9251, St. Louis, MO, USA), for diazotization reaction with nitrite content of the sample, and 40 mM N-(1-naphthyl) ethylenediamine dihydrochloride (Sigma Aldrich, 222488, St. Louis, MO, USA), to convert the resulting diazonium salt to a colorful measurable by spectrophotometry, azo die in an acidic environment of ortho-phosphoric acid. The TNF-alpha content was examined by an ELISA kit according to the manufacturer’s instructions (Invitrogen, BMS607-3, Waltham, United States), and the optical density was measured at 450 nm with an absorbance microplate reader (Biotek, ELx800, Winooski, VT, USA).

### 2.4. Preparation of Silk Fibroin Formulations

Silk fibroin was extracted and purified from *Bombyx mori* cocoons, generously provided by Professor J. L. Cenis (Instituto Murciano de Investigación y Desarrollo Agrario y Medioambiental-IMIDA, Murcia, Spain). Cocoons were washed, cut into small pieces, kept at 60 °C for 24 h for removal environment humidity, and degummed for 30 min in 0.2% (*w*/*v*) of sodium carbonate solution to remove the sericin coating. After being repeatedly rinsed in distilled water, the degummed silk was dissolved in 9.4 M LiBr in a 20% (*w*/*v*) silk to LiBr solution for three hours at 60 °C and dialyzed against distilled water with a SnakeSkin™ Dialysis tube with the cut-off size of 3.5 KDa (Thermofisher, 68035, Waltham, United States) [47]. The dialysis was changed daily until the electroconductivity was lower than 10 µS/cm. The clean extract of SF was frozen at −80 °C, lyophilized to isolate a solid powder, and stored at −20 °C until usage.

Silk fibroin-Rhodamine (SF-Rho) solutions were prepared as follows. First, SF powder was dissolved in NaHCO_3_ buffer (10 mg/mL) and kept with agitation for 20 min at room temperature. This solution was centrifuged and dissolved with Rhodamine B Isothiocyanate (Sigma-Aldrich, 283924, 0.6 mg/mL in dimethyl sulfoxide) in a proportion of 1:60 (Rhodamine solution: SF). To separate the unbound rhodamine, the SF-Rho solution was dialyzed against distilled water for four cycles of 12 h each. The resulting solution was lyophilized, and the powder was stored at −20 °C. SF or SF-Rho powders were resuspended in PBS at a 2% (*w*/*v*) concentration.

The gelification of different SF solutions was induced by sonication, as previously described [31]. Briefly, the SF solution at 2% (*w*/*v*) was poured within a thermally conductive mold and sonicated under constant intensity, time, and temperature (10% amplitude, 30 s, and 45 °C) using a Sonifier ultrasonic cell disruptor (Branson, S450D, Brookfield United States) connected to a 3 mm diameter tapered microtip. Reconstitution of SF with black ink (Pelikan, 201665, Berlin, Germany) was performed immediately after sonication at 1% (*v*/*v*) ink to SF solution concentration.

### 2.5. Preparation of Biomaterials Formulations

Collagen extraction and processing were performed as previously reported [48]. Briefly, type I collagen was obtained from rat tendons. The extracted tendons were collected in the PBS, transferred to acetone for 5 min, further cleaned in 70% isopropanol solution, and dissolved in 0.02 N acetic acid. The Collagen I solution was centrifuged, and the viscous supernatant was lyophilized. The resulting collagen powder was kept at −80 °C until further usage. Collagen hydrogels were obtained after dissolving the powder in 0.02 N acetic acid solution to a final concentration of 3 mg/mL for zymography studies or subsequent reconstitution on PBS at pH = 7 and black ink at 1% concentration for intracerebral injection.

Casein was extracted from the fresh bovine skimmed milk after the process of lactose separation by slowly adding 0.2 N hydrochloric acid until protein agglomeration. The solution was centrifuged, and the protein pellet was washed several times in distilled water and lyophilized. The resulting powder was dissolved in distilled water for zymography studies [49].

### 2.6. Proteolytic Activity on Substrate Gels

Zymography gels were conducted following previous methodologies [50]. Briefly, silk fibroin gels were fabricated at a concentration of 2 mg/mL. Parallel experiments were performed with gelatin (1.5 mg/mL), collagen (0.6 mg/mL), and casein (0.4 mg/mL) substrates. All gels were stabilized and polymerized in the presence of 8% Polyacrylamide gel (PAGE). The different substrates were incubated with a conditional medium derived from non-activated and activated microglia, proteinase K (Sigma Aldrich, P6556), or with recombinant Mouse/Rat MMP-2 (R&Dsystems, 924-MP, Minneapolis, MN, USA) and MMP-3 (R&Dsystems, 548-MM, Minneapolis, MN, USA). MMP-2 and MMP-3 were activated in the presence of 1mM of p-aminophenylmercuric acetate (Sigma Aldrich, A-9563, St. Louis, MO, USA) for 2 h at 37 °C. Protein samples were added to a sample buffer containing sodium dodecyl sulfate (SDS), glycerol, and bromophenol blue. Heating the samples was avoided to prevent protease inactivation, and electrophoresis was carried out under non-reducing conditions [51]. After electrophoresis (about 3 h at 110V at 5 °C), protein renaturation was promoted by washing the substrate gel with 2.5% of Triton X-100. Subsequently, the gel was incubated in Tris buffer pH of 7.4, 10 mM CaCl_2,_ and 0.02% sodium azide for 54 h at 37 °C. Afterward, the gels were stained with Coomassie brilliant blue.

### 2.7. Hydrogel-Microglia Interaction Studies

Collagen and silk fibroin hydrogels in a cylindrical shape (in mm, diameter: 4.0, height: 3.0; approximate volume ~35 μL) were incubated in a conditional medium derived from non-activated or activated (LPS) BV2 microglial cells. For positive control of hydrogel degradation, 0.6 Units/mL of proteinase K was dissolved in PBS and incubated with the biomaterial.

Hydrogel degradation was inferred by the analysis of wet weight, volume, and amount of β-sheet content of the overall structure by attenuated total reflectance–Fourier transform infrared spectroscopy (ATR-FTIR). The analysis of hydrogel wet weight, volume, and β-sheet content was performed at 1, 3, and 7 days after incubation with microglia-derived medium or proteinase K.

**Figure 1:**
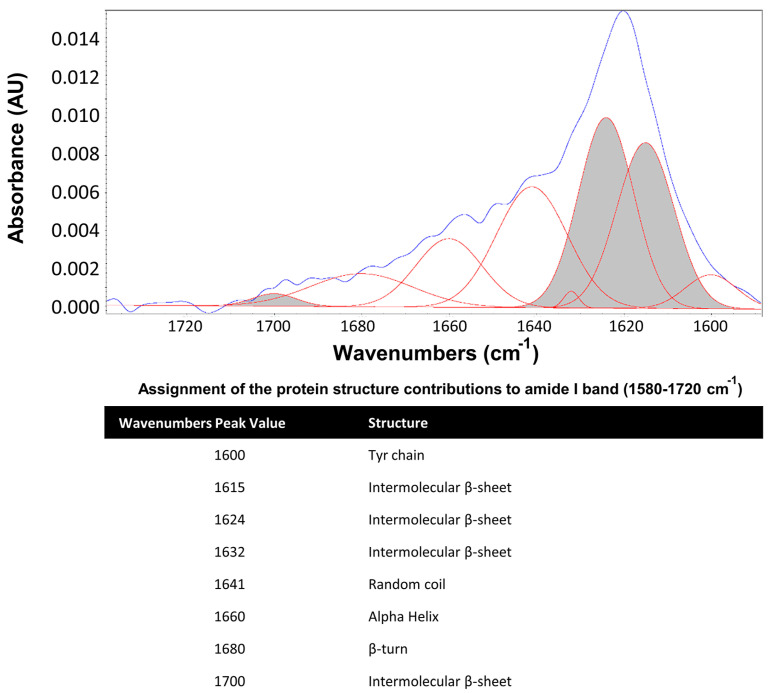
Secondary structure assignments of amide I band components in silk fibroin proteins. The amide I band region and the constituent resolved peaks are indicated as blue and red colors, respectively. The β−sheet area under the amide I band is in shaded gray.

The secondary structure content was analyzed in a Nicolet iS5 FTIR (Thermofisher, United States) with an ATR module to obtain the ATR-FTIR spectra protein in the range of 550–4000 cm^−1^ with 64 scans per spectrum and a resolution of 4 cm^−1^. The percentage of the β-sheet crystalline structure was estimated following a methodology previously used to determine the secondary structure of regenerated silk [23,52]. Briefly, the amide I region between 1600 and 1700 cm^−1^ was selected and fitted to Gaussian functions implemented in OMNIC 9 software (Thermofisher, Waltham, United States). To calculate the percentage of β-sheet content, the Gaussian area corresponding to the β-sheet structure (majorly the region of 1621–1637 cm^−1^; Figure 1 [23]) was divided by the total Gaussian fit area of all secondary structures and multiplied by 100 [52].

### 2.8. Mechanical Tests

Mechanical properties of SF and collagen hydrogels were assessed by compression tests on hydrogel cylinders performed in air. The compressive load was applied along the cylinder axis and measured with a balance (Precisa, XT220A, Dietikon, Switzerland) positioned on the lower supporting platform. The load was applied at a constant displacement rate of 1 mm/min. The cross-sectional areas were used to determine stress–strain (σ,ε) curves from force-displacement curves. Stress was calculated as the instantaneous force divided by the initial cross-sectional area, and strain as the displacement divided by the initial length. The elastic modulus and compressive strength were calculated using a custom code implemented on MATLAB (Math Works, Natick, MA, USA).

### 2.9. Stereotaxic Surgery and Biomaterial Injection

Silk fibroin was implanted in the caudate-putamen (striatum) following previous approaches reported by our group [31]. Briefly, after anesthesia with Ketamine/Xylazine, animals were stereotaxically injected with 5 microliters of a sonicated solution of SF, SF reconstituted with black ink (SF-Ink), or SF functionalized with rhodamine (SF-Rho). The different sonicated SF formulations (2% SF concentration in PBS) were infused unilaterally into the striatum at a rate of 1 μL/min, using a Hamilton syringe in the following coordinates from bregma: posterior 0 mm, laterally +2.0 mm, and ventrally 3.0 mm. An identical approach was used for collagen, which was injected (5 μL) in the striatum immediately after reconstitution with black ink at 1% concentration (*v*/*v*).

### 2.10. In Vivo Quantification of Silk Fibroin and Collagen Hydrogels

At 1, 7, and 14 days after SF hydrogel injection as well as 1 and 14 days after collagen hydrogel injection, the mouse brains were cut into coronal sections 1 mm thick and incubated with 4% paraformaldehyde (PFA) for 48 h. Images were acquired from both sides of each coronal section in a stereoscopic microscope (Leica, S6D, Wetzlar, Germany) coupled with a digital camera (MC170HD, Leica, Germany).

In mice implanted with SF-Ink or Collagen-Ink hydrogels, the amount of black matter for each slice of the brain was calculated by applying a color threshold to separate the gel from the surrounding tissue. The area occupied by the gel was calculated after the normalization of the image pixels with the corresponding scale and conversion of pixels into a SI mm^2^ scaling system. The distribution of the hydrogel area across the rostrocaudal axis was based on classical anatomic brain references in mice [53]. For an estimation of hydrogel volume, the two faces of each brain section were considered as individual pieces with a thickness of 0.5 mm. In mice implanted with SF-Rho hydrogels, the size occupied by Silk-rhodamine fluorescence per brain section was captured in a fluorescence chamber via an ImageQuant™ LAS 500 (Cytiva, Marlborough, MA, USA) system with a constant fluorescent exposure time.

### 2.11. Statistical Analysis

All of the plots and statistical analyses were performed within SPSS Statistics 26 (IBM, New York, NY, USA). The plotting and data analysis of mechanical studies were carried out by a custom code implemented on MATLAB R2020b (MathWorks, Natick, MA, USA). All the data are expressed as the means ± the standard error of the mean (SEM). A Student’s *t*-test for independent samples was used to evaluate significant differences in microglia survival, collagen, and silk-rhodamine volume. ANOVA with Tukey’s post hoc test was used to examine differences in the rest of the studies considering the following dependent variables: microglial parameters, percentage of β-sheet, weight, and volume; and the following independent variables: time and treatment (LPS, proteinase K, and mouse phenotype). Any *p* values below 0.05 were considered statistically significant.

## 3. Results

**Figure 2:**
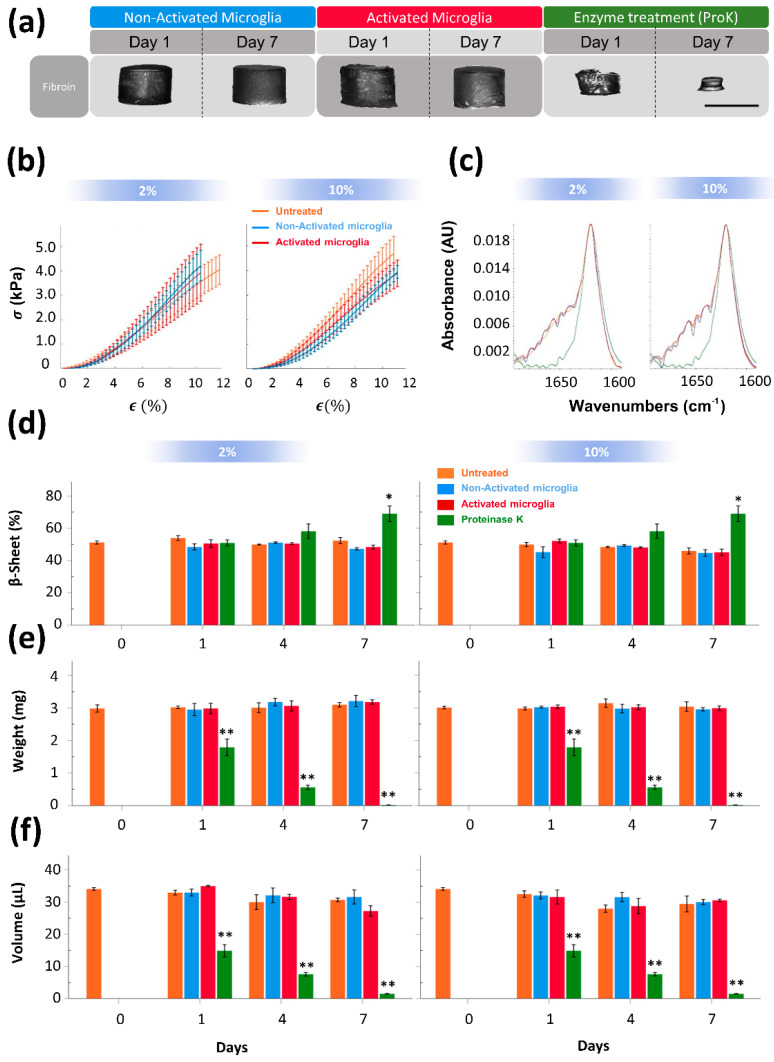
Silk fibroin hydrogels are resistant to activated BV2 microglial−derived medium. (**a**) Lateral view of SF hydrogels showing morphological changes at 1 and 7 days after incubation with a non−activated (blue) and activated (red) microglia−derived medium, or in the presence of proteinase K (green) (Scale bar 4 mm). (**b**) Stress−strain curves in untreated (orange line) and treated silk fibroin hydrogels seven days after incubation with non−activated (blue) or activated (red) microglia at low (2%) and high (10%) serum concentrations. The symbols σ and ε correspond with stress (in kPa) and strain (%) parameters, respectively. (**c**) Representative ATR−FTIR spectra of silk fibroin hydrogels incubated in untreated (orange line), non−activated (blue) or activated (red) microglia−derived medium, or in the presence of proteinase K (green). Note that degradation induced by proteinase K is associated with a reduction of absorbance in the regions corresponding to non-β−sheet structures. Percentage of β−sheet content (**d**), weight (**e**), and volume (**f**) in silk fibroin hydrogels across time after treatment. At least six samples were used per group. Data are shown as the means ± SEM. The asterisks denote the significant differences of different groups with respect to baseline (Day 0, untreated hydrogel). Statistical significance was determined via ANOVA and Tukey´s post hoc test (n = 6, * *p* < 0.05; ** *p* < 0.01).

Some studies have reported that silk fibroin hydrogels of 2% concentration showed mechanical properties in a range of nervous tissue [31,32]. Initially, in this study, the responsiveness of silk fibroin hydrogels (2% concentration) in contact with brain-resident microglia activated by lipopolysaccharide (LPS) was examined. In the microglial cell line BV2, LPS produced moderate cell mortality and induced significant changes in cell morphology as BV2 extended more cellular processes (Figure S1). In addition, LPS reduced the rate of proliferation and increased the secretion of the inflammatory molecules TNF-α and NO (Figure S1). These observations are indicative that, in our hands, LPS incubation polarized microglia towards a pro-inflammatory phenotype. In vitro, during a seven day analysis, the morphology, mechanical, and structural properties of SF hydrogels were relatively well preserved when in contact with LPS-activated (inflammatory) and non-activated microglia (Figure 2a–c). In contrast, SF hydrogels incubated with the fungal proteinase K showed drastic morphological changes (Figure 2a, right) and a reduction of the amide I region corresponding to non-β-sheet secondary structure (Figure 2c and Figure S2) that reflected the degradation of this biomaterial after coming in contact with this enzyme. Over time, the percentage of β-sheet content (Figure 2d and Figure S2), weight (Figure 2e), and volume (Figure 2f) of SF hydrogels in contact with the culture medium, non-activated, or an activated microglia-derived medium was similar. Meanwhile, proteinase K produced a gradual reduction in SF hydrogel weight and volume, while in every analyzed time point, the percentage of β-sheet in the remaining hydrogel was stable or even superior seven days after proteinase K incubation, this time coinciding with the point of maximal degradation. Furthermore, additional experiments were performed with SF hydrogels incubated with an equivalent volume of a microglia medium obtained from activated microglial cells five times more concentrated than in this previous study, thus leading to an expected higher concentration of inflammatory factors and proteases in contact with the biomaterial. However, the weight, β-sheet content (%), or mechanical properties did not considerably change in these more extreme conditions (Figure S3).

**Figure 3:**
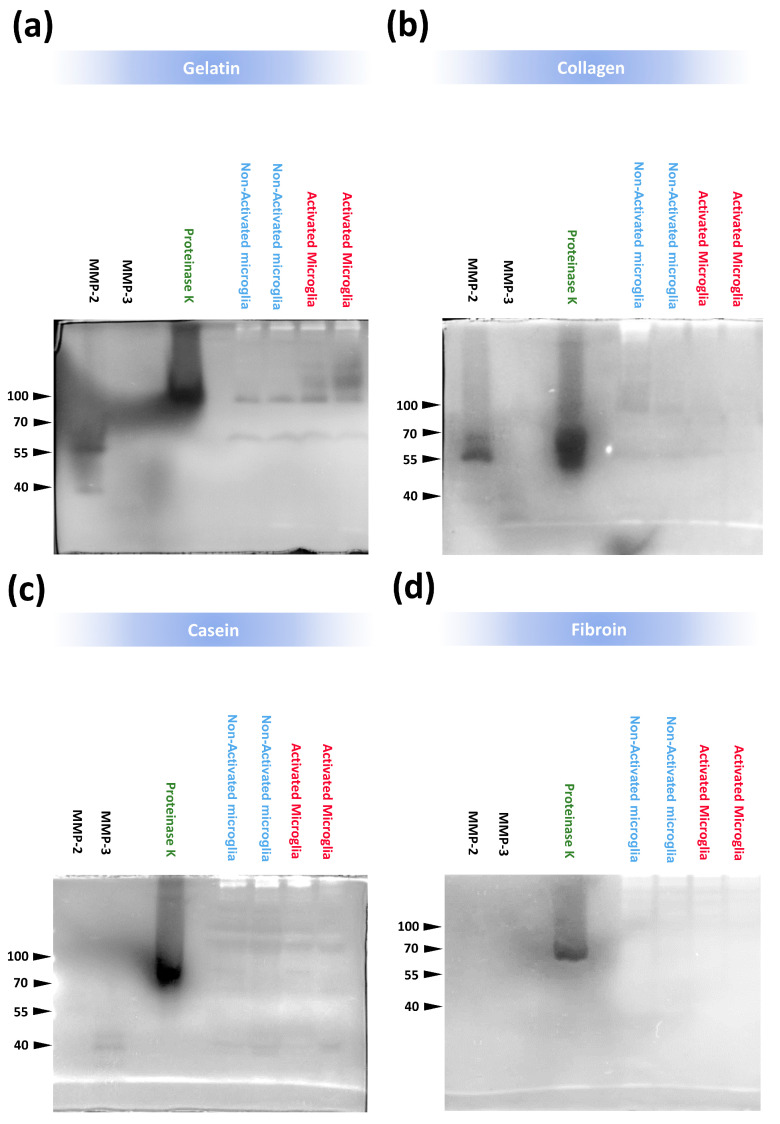
Enzymatic digestion of silk fibroin gels in contact with proteolytic enzymes and BV2 microglial−derived medium. Zymography analysis to examine the enzymatic activity of MMP−2, MMP−3, proteinase K, and conditioned media from non−activated and activated BV2 microglia cells on different biomaterial substrates: (**a**) gelatin, (**b**) collagen, (**c**) casein, and (**d**) silk fibroin. Note that all the materials were digested with proteinase K (molecular weight under non−denaturing conditions: ≈ 70 kDa, except for gelatin with a molecular weight above 100 kDa). Gelatin and collagen were digested with MMP−2 (molecular weight ≈ 55–60 kDa) and casein slightly with MMP−3 (molecular weight ≈ 40 kDa), while degradation of gelatin and collagen with MMP−3 was not evident. In the blot lanes that correspond to BV2 microglial−derived medium, it was evident the presence of different protein bands with intense or moderate enzymatic activity in all materials tested, with the exception of silk fibroin, which only was clearly digested with proteinase K. The images shown are representative zymogram analyses performed with a minimum of 3 blots for each biomaterial tested (gelatin, collagen, casein, and silk fibroin).

Zymography techniques have been used previously to examine the proteolytic activity of different MMPs over different substrates [54], but this approach is novel with respect to the use of SF as a target substrate. The performance of zymography studies was important in obtaining additional mechanistic aspects linked with the activity of several MMPs in contact with SF. While proteinase K degraded all the material tested, including gelatin (Figure 3a), collagen (Figure 3b), casein (Figure 3c), and silk fibroin (Figure 3d), MMP-2 degraded gelatin and collagen, and MMP-3 slightly degraded casein. However, SF was not degraded by MMP-2 or MMP-3 (Figure 3d). In addition, the conditional medium derived from non-activated or activated BV2 microglia revealed traces of degradation in collagen, gelatin, and casein gels, which were probably associated with the activity of several proteases present in this medium. In contrast, signs of SF degradation induced by the microglia-derived medium were not evident. These in vitro studies supported the resistance to degradation of SF hydrogels in contact with inflammatory microglia since this biomaterial format apparently did not show significant structural or mechanical alterations or enzymatic digestion by several MMPs.

**Figure 4:**
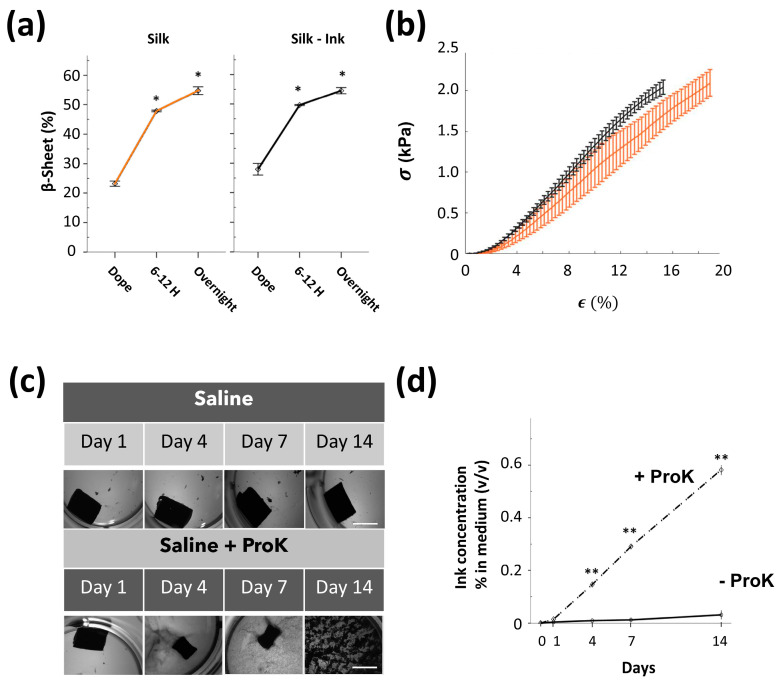
Silk fibroin hydrogels reconstituted with carbon ink showed unmodified gelation time course and equivalent mechanical properties compared to intact silk fibroin hydrogels. (**a**) Percentage of β−sheet in intact silk (orange) and silk reconstituted with ink (SF−ink) (black), in dope (initial SF protein dope before sonication), 6–12 h after sonication (timepoint of biomaterial gelation), and overnight (after incubation at 37 °C). Significant differences were analyzed with ANOVA followed by Tukey´s post hoc test compared to dope (three samples were used per group and time point, before and after sonication). (**b**) Stress–strain (σ, ε) curves of SF (orange) and SF reconstituted with carbon ink (black). (**c**) Representative top views of SF hydrogels reconstituted with ink and incubated in PBS (saline solution) or in the presence of proteinase K during 14 days of incubation. Note the progressive deterioration of SF−ink hydrogels and delivery of ink to the medium in the presence of proteinase K (scale bar 4mm). (**d**) Quantification of the amount of released ink in the solution in the presence and absence of proteinase K. Statistical significance was calculated by a two−way ANOVA with Tukey’s post hoc test compared to day 0 (at least three samples were used per group and temporal point). Data are shown as the means ± SEM. The asterisks denote significant differences between different groups with respect to dope (panel **a**) or basal condition (Day 0 in panel **d**); * *p* < 0.05; ** *p* < 0.01.

The studies of the interaction between SF and the BV2-microglia-derived medium and MMPs were affected by limitations related to the simplicity and dissection of the inflammatory context, which in the pathological brain comprises additional inflammatory cell populations; for example, reactive astrocytes and peripheral neutrophils and macrophages, with different dynamics of cell recruitment and activation. Therefore, we wondered whether the durability of this SF biomaterial could be affected in vivo in a more real inflammatory context induced by sudden injury or neurodegeneration. For in vivo evaluation and the improvement of the contrast visualization of SF in the brain parenchyma, formulations of SF reconstituted with carbon black ink were fabricated (SF-ink). Carbon black ink is tolerable in animals and has been used to visualize cerebral vasculature [55]. Before undertaking the in vivo studies, the gelation process occurred in vitro in a similar way between SF and SF-Ink (Figure 4a), reaching equivalent stiffness properties (Figure 4b). In a saline solution, these SF-ink gels were very stable, and no morphological changes occurred over two weeks. This stability constituted an essential pre-requirement for further in vivo quantification of SF deposits in the brain parenchyma inferred from the location of a quantifiable binding of SF and ink. By contrast, the in vitro stability of SF-Ink hydrogels was disrupted by proteinase K, which induced a gradual degradation of SF linked with a progressive biomaterial shrinkage and release of ink to the medium (Figure 4c,d). Because traces of ink rapidly and progressively disappear after intracerebral injection of ink alone (Figure S4), if SF degradation happened in vivo, one would expect the disappearance of ink from the degraded hydrogel area.

**Figure 5:**
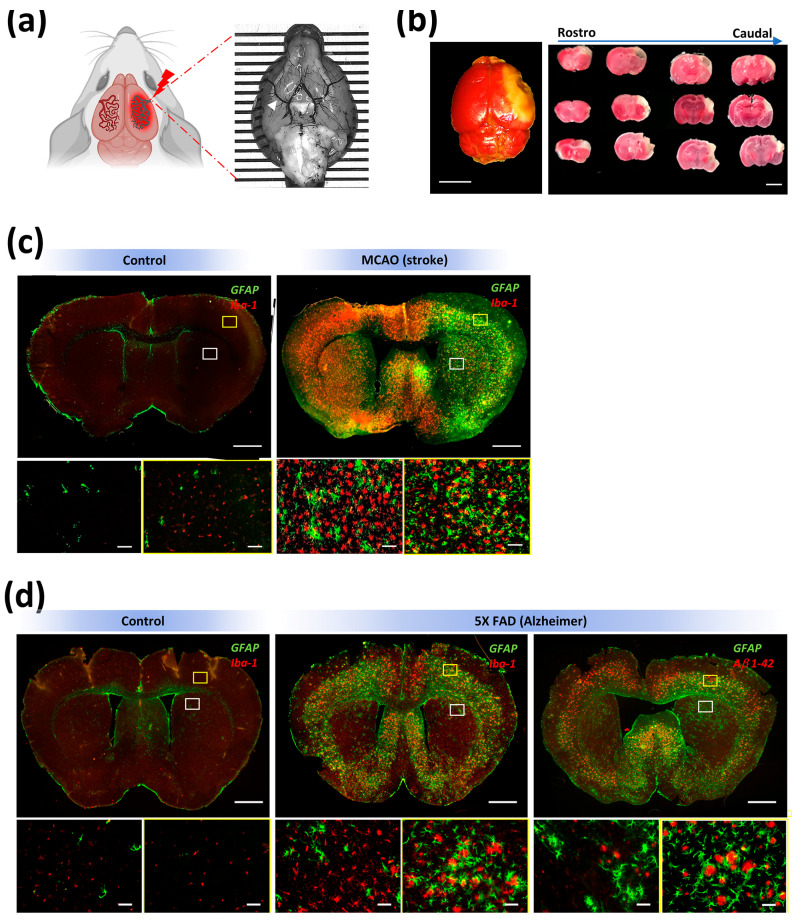
Main neuroinflammatory hallmarks found in pathological models of MCAO (stroke) and 5× FAD (Alzheimer’s). (**a**) Left panel, cartoon illustrating the procedure of middle cerebral artery occlusion (MCAO). Right panel, ventral view of a mouse brain stained with black ink to label the cerebral vasculature. Picture and graphical abstract were designed with BioRender software with further modifications. In the surgical procedure (MCAO), this artery (white arrowhead) is occluded at the distal level with respect to the Willis polygon. (**b**) Left panel, the MCAO approach produces a brain infarction (white region after TTC staining) mostly restricted to somatosensory areas, while motor regions were less affected (scale bar 3 mm). Right panel, coronal brain sections showing the location of an infarcted area across the rostrocaudal axis (scale bar 3 mm). (**c**) Representative images of coronal brain sections at low (scale bar 1 mm) and high (scale bar 100 μm) magnification to immunophenotypically identify reactive astrocytes (GFAP, green) and microglia/macrophages (Iba−1, red) in control (left panel, no stroke) and MCAO (right panel, stroke) mice. Note the high density of astrogliosis and microgliosis, especially in the cortex and striatum in stroke animals. (**d**) Representative images of coronal brain sections at low (scale bar 1 mm) and high (scale bar 100 μm) magnification to immunophenotypically identify reactive astrocytes (GFAP, green) and microglia/macrophages (Iba−1, red) in control (left panel, no Alzheimer’s) and 5× FAD (middle panel, Alzheimer’s) mice. Note the high density of reactive astrocytes and especially microglia in the cortex and striatum of Alzheimer’s animals. In the right panel, reactive astrocytes (GFAP, green) surround amyloid−β deposits (Aβ1−42, red) located in the cortex and in lower extension in the striatum.

We tested the stability and possible biodegradation of SF hydrogels implanted in the striatum (caudate–putamen) using two in vivo pathological models. The first was a very clinically relevant stroke model produced by the permanent occlusion of the middle cerebral artery (MCAO). In mice, the MCAO model (Figure 5a) produced cortical infarctions affecting the somatosensory areas and the motor regions in lower extension (Figure 5b). In our hands, the MCAO model also produced severe neuroinflammation linked with intense microgliosis and astrogliosis in the cortex and striatum of the infarcted hemisphere (Figure 5c). The second pathological model mimicked the progressive neurodegeneration and inflammation observed in Alzheimer’s disease (AD). In our study, AD (5× FAD) mice showed amyloid-β plaques in several brain regions, such as the subiculum, cortex, and hippocampus, although amyloid-β deposits content was lower in the striatum (Figure 5d, right). Likewise, activated microglia and astrogliosis were detected in the cortex and in a minor extension in the striatum of 5× FAD mice (Figure 5d, middle).

**Figure 6:**
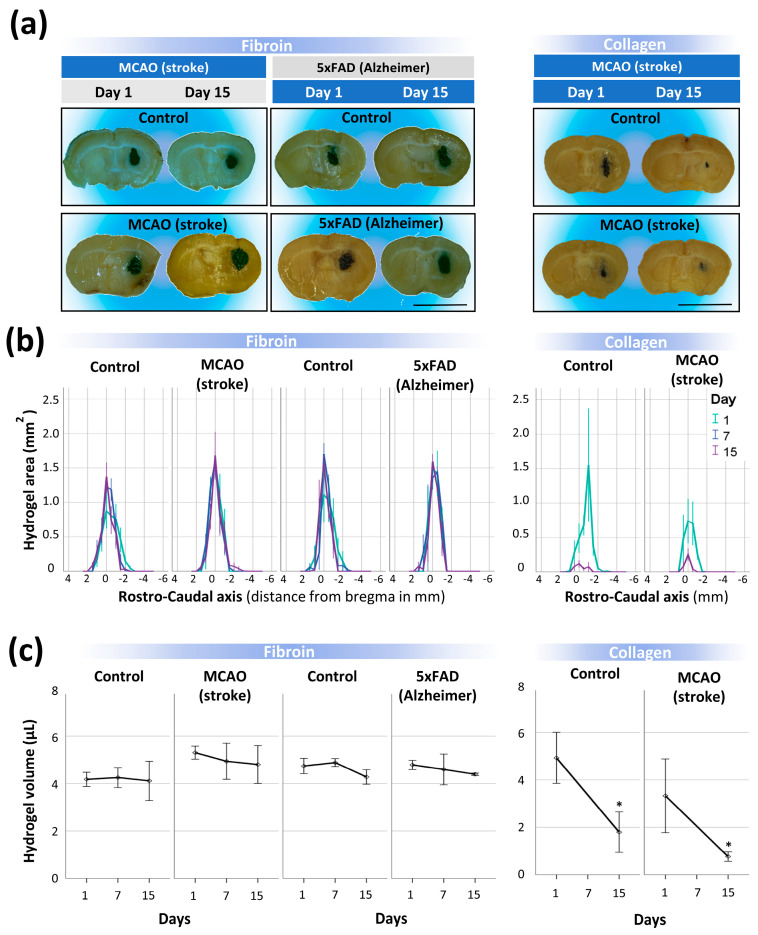
Degradability of silk fibroin hydrogels implanted in the inflammatory brain. (**a**) Left panel, representative coronal brain sections of healthy, MCAO (stroke) and 5× FAD (Alzheimer´s) animals at 1 and 15 days after implantation of silk fibroin hydrogels reconstituted with ink (scale bar 5mm). Right panel, brain coronal sections corresponding with healthy and stroke mice implanted with collagen hydrogels. (**b**) Plots showing the silk fibroin (left panel) or collagen (right panel) hydrogel area across the rostrocaudal axis at different time points after implantation (1, 7, and 15 days). On the x−axis, zero indicates the anatomic reference bregma, which is coincident with the point of biomaterial injection. A minimum of 6−10 and 3−6 silk fibroin-injected mice were used per temporal group in the stroke and Alzheimer’s groups, respectively. A minimum of 5−9 collagen-injected mice were analyzed per group and temporal point. (**c**) Estimation of silk fibroin hydrogel volume in the brain of healthy, stroke, and Alzheimer’s mice over time after injection. In the right panel, note the substantial decay of collagen hydrogel volume over time after injection in control (no stroke) and stroke animals. Data are shown as the means ± SEM. Statistical significance was examined by a Student’s *t*−test in the collagen studies and ANOVA followed by Tukey’s post hoc test for silk fibroin assays. The asterisks denote significant differences between different time points; * *p* < 0.05.

An in situ gelling silk fibroin hydrogel reconstituted with ink was injected into the striatum of healthy, MCAO (stroke), and 5× FAD (Alzheimer’s) mice. Histological studies from different brain coronal sections showed that the area occupied by the SF deposits across the rostrocaudal axis was relatively stable in the range of 1 (short-term) to 15 (mid-term) days after injection in both non-pathological and pathological animals (Figure 6). The quantification of total volume in the whole brain was similar between groups and relatively close to the injected volume (Figure 6a-left, Figure 6b-left, and Figure 6c-left). Although collagen hydrogels reconstituted with carbon ink showed very good in vitro stability disrupted only by proteinase K (Figure S5), collagen hydrogels rapidly disappeared in vivo in healthy and stroke mice (Figure 6a-right, Figure 6b-right, and Figure 6c-right).

**Figure 7:**
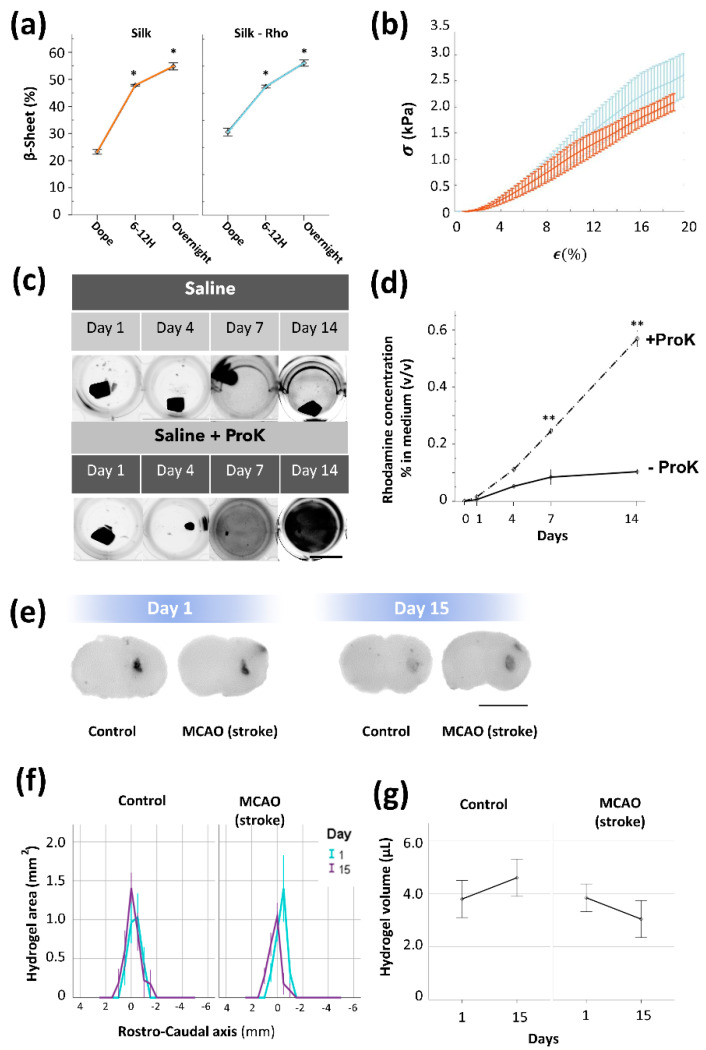
Degradability of silk fibroin hydrogels functionalized with rhodamine (SF-Rho) after stroke. (**a**) Percentage of β−sheet in intact silk (orange) and silk functionalized with rhodamine (blue), in dope (the initial SF protein dope before sonication), 6−12 h after sonication (time point of biomaterial gelation), and overnight (after incubation at 37 °C). (**b**) Stress–strain curves in non−modified SF (orange) and SF functionalized with rhodamine (SF−Rho) (blue). (**c**) Representative top views of silk fibroin hydrogels functionalized with rhodamine in the presence of PBS (saline solution) or proteinase K during 14 days of incubation. Images were captured in a fluorescent chamber. Note the progressive deterioration of SF−Rho hydrogels in the presence of proteinase K, with increasing fluorescence intensity in the medium (scale bar 10 mm). (**d**) Quantification of rhodamine released in solution in the presence or absence of proteinase K. (**e**) Representative coronal brain sections of healthy and MCAO (stroke) animals, at 1 and 15 days after implantation of SF−Rho (scale bar 5 mm). (**f**) Plots showing the hydrogel area across the rostrocaudal axis at 1 and 15 days after silk fibroin implantation (a minimum of four mice were used per group and temporal point). (**g**) Estimation of silk fibroin hydrogel volume in the brains of healthy and stroke mice over time after injection. Data are shown as the means ± SEM. In (panels **a** and **d**), the statistical significance was calculated by ANOVA followed by Tukey’s post hoc test. At least three samples were used per group and temporal point. The asterisks denote significant differences between different groups with respect to dope (panel **a**) or baseline condition (Day 0 in panel **d**); * *p* < 0.05; ** *p* < 0.01. In (panel **g**), statistical significance was examined by a Student’s *t*−test.

Additional studies were performed to analyze the stability of silk fibroin from a distinct perspective using a different biomaterial formulation. In this case, we functionalized SF with the fluorescent molecule rhodamine (SF-Rho). In vitro after gelation (Figure 7a,b), SF-Rho showed good compartmentalization of rhodamine, which was impaired only after enzymatic digestion with proteinase K (Figure 7c,d). In vivo, although the fluorescent intensity decayed between Day 1 and 15 (Figure 7e), the area occupied by the SF-Rho hydrogel and the total volume did not significantly differ between non-stroke and stroke animals (Figure 7f,g). Collectively, these in vivo results quantitatively supported the short- and mid-term stability of regenerated SF hydrogels implanted in the brain tissue, which was resistant to molecular- and cellular-based inflammatory components that, in contrast, rapidly disrupted collagen hydrogels in the cerebral tissue.

## 4. Discussion

Although there have been considerable advances in the applicability of synthetic and natural materials for better control of drug delivery and stem cell engraftment in different pathologies, in a large majority of preclinical trials, there is a lack of information on how different materials respond when in contact with a pathological environment. Comprehensive studies on the stability of biomaterials-based products are crucial as part of the regulatory path to perform clinical trials in patients. Better knowledge of this may redirect our therapeutic designs for screening biomaterial formulations and formats that have more adequate profiles depending on specific intervention windows.

Brain-resident microglia constitute the main component of innate immunoresponse in the CNS. After brain injury, inflammatory microglia and peripheral leukocytes extend the damage and enhance neurodegeneration by producing distinct pro-inflammatory factors and reactive oxygen species, such as nitric oxide, TNF-alpha, or IL-1β [56,57,58,59]. In addition, inflammatory microglia produce and overexpress a wide spectrum of MMPs and other enzymes that degrade ECM proteins, adhesion molecules, and cell surface receptors [60]. The increased secretion of pro-inflammatory factors by inflammatory microglia contrasts with reduced phagocytosis, which is mostly present in non-inflammatory microglia [61], which can directly engulf and phagocytose ECM-derived components for brain tissue remodeling and repair [62,63]. Microglia cells are also involved in the acute and chronic foreign body reaction (FBR) responses against implanted materials [64,65]. In addition, the acute phase of FBR is characterized by the early migration of peripheral neutrophils to the area of the implant [37,64]. These neutrophils produce a set of pro-inflammatory signals, induce the secretion of proteolytic enzymes, and contribute to the cellular accumulation of reactive oxygen species. Subsequently, these pro-inflammatory molecules affect vascular permeability and enhance monocyte recruitment to the site of the implant that will differentiate into macrophages [64]. This complex inflammatory environment is also intensified by the activation of microglia with the ability to secrete additional inflammatory cytokines and the production of reactive oxygen intermediates [65].

In vitro, microglia can be polarized towards inflammatory profiles with different stimuli such as oxygen/glucose deprivation or the anti-viral molecule interferon. Moreover, the Gram-negative bacterial endotoxin LPS is also a well-known inductor of microglia activation towards inflammation [66]. In our study, SF hydrogels were initially exposed to the secretome of LPS-stimulated BV2 microglia. This medium contained a set of pro-inflammatory factors such as NO or TNF-alpha, but in addition, LPS-stimulated BV2 cells showed overproduction of several MMPs members, such as MMP-3, MMP-8, and MMP-9 [67]. Additional studies have shown that LPS increased the expression of MMP-2, MMP-9, MMP-12, and MMP-14 in the microglia of both humans and mice [60,68]. Upon in vitro incubation with this secretome-derived inflammatory microglia, SF hydrogels remained intact and were resistant to enzymatic digestion, at least during one-week analysis. This observation contrasted with the marked sensitivity detected in our study for non-SF biomaterials exposed to MMPs (MMP-2 and MMP-3) and the conditional medium derived from BV2 cells. This evidence, in combination with the in vivo results reported in this study, suggests that SF is resistant to the proteolytic degradation induced by the acute stage of FBR (as a response to the implant) and by the neuroinflammatory response induced in every respective pathological model.

A limitation of the in vitro design is that it was not examined the responsiveness of SF hydrogels in direct contact with inflammatory BV2 microglial cells or other inflammatory cells, such as peripheral neutrophils and macrophages, which are recruited to the sites of inflammation in response to brain injury. Although microglia are ontogenetically and functionally related to macrophages and circulating myeloid cells, microglia show elevated longevity, self-renewal capacity, and different transcriptomic profiles [69,70]. These differential facts, in addition to the induction of astrogliosis, reflect the exclusive particularity of cerebral tissue in relation to non-brain tissues and that the stability of silk fibroin in response to neural injury and inflammation cannot be initially anticipated from previous studies that have analyzed the degradability of SF in inflammatory non-brain environments.

Due to the reductionist approach of the in vitro assays, in this study, an in vivo scheme that materialized a complex but closer condition to the neuroinflammatory context was included. This complex condition is highlighted in strokes due to the specific temporal pattern of non-inflammatory and inflammatory microglia activation in the cortex and striatum [61]. As early as 24 h after stroke, a transient activation of non-inflammatory microglia that peaks after one week and strongly declines two weeks after injury is induced [61]. Such a time period is equivalent to the definitive transition from the acute to the chronic stage of FBR. During chronic FBR, microglia polarizes toward a non-inflammatory phenotype. However, this stage is generally characterized by the generation of polynucleate macrophages (foreign body giant cells) and the gradual formation of a fibrotic capsule around the implant [37]. In this context, a remarkable feature of SF is its ability to reduce the formation of the fibrotic capsule, and it has been reported the advantage of using silk fibroin for silicon implants to reduce the capsule thickness [71].

During the acute phase of the stroke, microglia is polarized towards inflammatory phenotypes (at 48–72 h after ischemia), and this inflammatory population is even four times higher two weeks after injury. In addition, the recruitment of different types of peripheral leukocytes towards the lesioned hemisphere, mostly neutrophils and macrophages, occur in the first days after brain ischemia [57]. During this complex and particular timeframe, which corresponds to the acute and the beginning of subacute stroke phases, in our study, SF was highly resistant to in vivo degradation, an observation that contrasted with the rapid degradation observed for cerebrally implanted collagen hydrogels in both healthy and stroke animals. Previous studies have shown that ECM hydrogels implanted in the stroke cavity showed 80% degradation 14 days post-implantation [72], a fact probably explained by the high sensitivity of ECM proteins, including collagen, to MMPs secreted by different inflammatory cells. In this aspect, the natural biodegradability of ECM-based designs such as collagen hydrogels may be very relevant for a short therapeutic window, where a therapeutic timeframe inferior to the stability of the material itself is acceptable. Microglia also play a significant role in neurodegeneration progression. In Alzheimer’s disease, for example, microglia surround and establish a narrow contact with amyloid-β deposits. Inflammatory microglia have been related to plaque growth [73] and very recently to amyloid-β pathology propagation and disease progression [74]. The neuroinflammatory response triggered by microglial cells can also lead to the activation of astrocytes that similarly surround the accumulations of amyloid-β while constantly secreting pro-inflammatory factors [75] and matrix remodeling enzymes such as MMP-2 and MMP-9 [75,76,77]. In this complex neurodegenerative and inflammatory environment, SF hydrogels were relatively stable as in the stroke model.

Although in our study, the results from both pathological models anticipated a strong resistance of this material to degradation by proteases, previous studies have shown that SF is degraded by α-chymotrypsin from the bovine pancreas [78], collagenase from the bacterium *Clostridium histolyticum*, proteinase XXI from *Streptomyces* sp. [79], and similar to in our study, SF was degraded by proteinase K [80]. However, SF has been considered a non-degradable material by the United States Pharmacopeia (USP) due to the preservation of mechanical properties in SF sutures even after more than 150 days post-implantation [81]. Furthermore, SF is also relatively stable when exposed to several members of the inflammatory MMPs family [80,82]. In contrast, other evidence has shown the ability of SF to degrade in vivo. For example, in the context of CNS, it has been reported the SF can be degraded after intracerebroventricular injection [38]. Furthermore, a non-significant tendency toward the reduction of SF volume was seen in patients with postoperative hematomas, where patients showed a reduction in the concentration of silk sponges implanted either intramuscularly or subcutaneously [11,83]. The subcutaneous biodegradation of SF-based materials has also been confirmed using ultrasound with SF-PEG hydrogel formulations [10]. This evidence raises questions regarding the stability of SF depending on the target tissue/organ. Perhaps, these discrepancies might come from the use of SF with various distributions of molecular weights, different proportions of secondary structures found in the different formats, and variations in the degumming protocols that can lead to contamination with sericin traces, as SF scaffolds showed more degradation with higher content of sericin [84]. Furthermore, it has been reported that the proteolytic degradation of silk is dependent on the type of enzyme and biomaterial structure [78,80]. For example, the enzymatic treatment of silk hydrogels, especially with proteinase K, can be expected to primarily affect the α-helix and random coil structures, increasing the concentration of the β-sheets in the hydrogel, a phenomenon that was observed in our in vitro study (Figure 2c,d).

As far as we know, the strategy of hydrogel reconstitution with carbon ink as a contrast agent has not been previously used to analyze the stability and degradability of hydrogels in different tissues, including the nervous system. Alternative strategies for the measurement of decellularized-ECM hydrogels in the brain have been based on immunochemistry against different biomaterials [72,85]; however, our strategy might be optimal for detecting biomaterial formulations based on synthetic materials or natural materials such as SF, which are poorly immunogenic. The application of non-invasive in vivo imaging procedures such as magnetic resonance imaging (MRI) or micro-computed tomography are costly procedures that are also hampered by the low contrast of many biomaterials and formulations [86]. In our case, SF hydrogels reconstituted with gold nanoparticles were detected by micro-computed tomography in the non-pathological brain, showing, in agreement with this study, great stability for at least two weeks after implantation (non-published data). Although previous studies have reported the use of ultrasmall superparamagnetic iron oxide nanoparticles to subcutaneously detect silk fibroin/hydroxyapatite scaffolds [87], for longitudinal imaging, the use of high concentration of nanoparticles to enhance the contrast resolution of SF and other biomaterials raises concerns of toxicity that could compromise functional outcomes during preclinical evaluations [88,89,90].

The great stability and poor degradation of SF hydrogels in the pathological brain might perhaps be explained by the biochemical, rheological, and inductive properties of this material. For example, it is widely accepted that SF is a biologically inert biomaterial, and due to not yet deciphered mechanisms, this material does not evoke a significant immunological activation [37,91,92]. The aminoacidic sequence and particular structure of this material that camouflages antigenic determinants blocking immune recognition might also prevent the enzymatic digestion of physiological proteases. Although MMPs cleavage sites have been identified in the amino acid sequence of SF, they are not widely distributed in the SF backbone. Perhaps, such inertness can prevent the recognition of the SF hydrogel surface by the different molecular (for example, complement system) and cellular components triggered by the FBR during the acute stage [37]. Interestingly, Huang and colleagues reported a fast degradation of transgenic SF that was previously modified with additional MMP-2 cleavage sites in the SF heavy chain [93]. By contrast, collagen deposits are shown to have a good interaction with different molecules and cells through different mediated-adhesion proteins while eliciting a moderate immune response [37]. Consequently, collagen biomaterials have been found in vivo to promote cell infiltration and invasion towards this biomaterial during acute FBR [37,94,95]. Thus, it has been widely reported the fast in vivo degradation of collagen implanted in different tissues [37,96,97]. The rheological properties furthermore could explain the more favorable in vivo degradation of collagen in relation to SF. Notably, the concentration of collagen used in this study was 10 times lower than SF to reach similar mechanical properties in both materials; thus, collagen provided lower material content per equal volume of injection for degradation. A less dense structure of collagen might provide a bigger network and channels for cellular and accelerated biodegradation. However, this theoretical model contrasts with the results of Siavashani and colleagues, where they found a reduction in the pore size with increasing sericin content and higher rates of degradation of SF scaffolds, an observation that they attributed to the amorphous and highly hydrophilic nature of the sericin molecule [84]. The inductive properties of SF are related to the capacity of SF to alleviate inflammatory and oxidative stress responses [26]. Specific SF-derived peptides such as Brain Factor 7 (BF7) promote neuroprotection through a reduction of reactive oxygen species content [98], and SF has shown anti-inflammatory properties in a mouse model of ear edema [18], possibly due to the inhibition of Mitogen-activated protein kinases (MAPKs) [99]. As MAPKs/NF-κB signaling is an important pathway that regulates the expression of inflammatory mediators in macrophages and microglia, perhaps SF and specific sequences such as BF7 can produce an inductive anti-inflammatory environment that partially shields the activation of invasive immune cells, attenuating or slowing down the in vivo biodegradation of SF hydrogels.

## 5. Conclusions

In this study, we expanded our view of the integrative capacity of silk fibroin for central nervous system applications. By using two different in vivo models of cerebrovascular pathology and neurodegeneration, which are linked with neuroinflammation of variable intensity and time course, using new quantification methods, the short- and mid-term biodegradability of this biomaterial in the healthy and pathological brain was studied for the first time.

In comparison with other natural materials such as collagen, which seems more appropriate for smaller therapeutic windows, our results support the high stability of silk fibroin even under extreme inflammatory conditions, which makes this biomaterial and format an ideal vehicle for the sustained release of cells and different molecules, such as neurotrophic factors for the induction of brain neuroplasticity, for treatments that require longer intervention windows.

Although SF is an innocuous biomaterial and shows high compatibility with the nervous system [30,31,32], the non-degradable properties of SF and the impossibility of eliminating the biomaterial once the therapeutic effect has concluded might constitute a limitation (for example, imposing mechanical restrictions for spontaneous tissue remodeling) that should be addressed in future studies. A possibility in this context is the design of new SF polymers; for example, genetically modified silk to enhance its sensitivity to MMPs and other physiological proteases [93] or the use of mixed formulations of degradable biomaterials with non-degradable SF [100,101]. These engineering strategies will advance our knowledge of more optimized designs with the precise tuning of SF degradability for better adaptation to specific therapeutic time windows in the treatment of non-cerebral and cerebral pathologies.

## Data Availability

The ATR-FTIR spectra files used in this study are available (Open Access) at Mahdi Yonesi, Milagros Ramos, Carmen Ramirez-Castillejo, Rocio Fernández-Serra, Fivos Panetsos, Adrian Belarra, Margarita Chevalier, Francisco J. Rojo, José Pérez-Rigueiro, Gustavo V. Guinea and Daniel González-Nieto Database from Resistance to Degradation of Silk Fibroin Hydrogels Exposed to Neuroinflammatory Environments [Data set]. Zenodo.org. 2023. https://doi.org/10.5281/zenodo.7921117 (accessed on 15 May 2023). The remaining data of this study are available from the corresponding author upon reasonable request.

## Supplementary Materials

The following supporting information can be downloaded at: https://www.mdpi.com/article/10.3390/polym15112491/s1, Supplementary methods; Table S1: Number of animals used in the intracerebral injection studies; Figure S1: Analysis of microglia viability; Figure S2: ATR-FTIR spectra of SF hydrogels across time after incubation with different medium conditions and proteinase K; Figure S3: In-vitro degradation of silk fibroin hydrogels in response to microglia cells seeded at high concentrations; Figure S4: In vivo clearance of black ink after cerebral injection; Figure S5: In vitro stability of collagen hydrogels.
