# Supplementary material for: Resistance to Degradation of Silk Fibroin Hydrogels Exposed to Neuroinflammatory Environments
Source: Polymers (Basel). 2023 May 28;15(11):2491. doi: 10.3390/polym15112491 (PMC10255612; doi:10.3390/polym15112491)
Supplement: Supplementary file 1 [file polymers-15-02491-s001.zip › polymers-2338253-supplementary.pdf]

SUPPLEMENTARY MATERIAL

Resistance to Degradation of Silk Fibroin Hydrogels Exposed to Neuroinflammatory Environments

Mahdi Yonesi, Milagros Ramos, Carmen Ramirez-Catillejo, Rocío Fernández-Serra, Fivos Panetsos, Adrián Belarra, Margarita Chevalier, Francisco J. Rojo, José Pérez-Rigueiro, Gustavo V. Guinea and Daniel González-Nieto

The supplementary material consists of:

Supplementary methods

Table S1- Number of animals used in the intracerebral injection studies

Figure S1- Analysis of microglia viability

Figure S2- ATR-FTIR spectra of SF hydrogels across time after incubation with different medium conditions and proteinase K

Figure S3- In-vitro degradation of silk fibroin hydrogels in response to microglia cells seeded at high concentrations

Figure S4- In vivo clearance of black ink after cerebral injection

Figure S5- In vitro stability of collagen hydrogels

Supplementary methods

1. Immunohistochemistry and histological studies

Cortical infarction in MCAO mice was examined by triphenyl tetrazolium chloride (TTC) staining [1]. At 24-48 h after stroke, mice were sacrificed and the brains were extracted, cut into 1 mm thick coronal sections, and incubated with a freshly prepared solution containing 1% TTC in PBS. Incubation was performed at room temperature for 10 min protected from the light. Brain inflammation was examined in stroke and 5xFAD mice transcardially perfused with 4% paraformaldehyde (PFA) in PBS. Brains were soaked in 30% D-sucrose and cut in coronal sections (30 μm) using a Cryostat Microm HM550 freezing microtome (Thermofisher). Brain sections from both pathological models were incubated with primary antibodies against the Glial Fibrillary Acidic Protein (GFAP) to label reactive astrocytes (Thermofisher Scientific, 13-0300), and the ionized calcium-binding adapter molecule 1 (Iba-1) to label microglia/macrophages (Abcam, ab178846). GFAP and Iba-1 primary antibodies were detected through fluorescently conjugated secondary antibodies against rat (712-546-153, Jackson ImmunoResearch) and rabbit (111-165-003, Jackson ImmunoResearch) respectively. Beta-amyloid plaques were detected with a primary antibody against Aβ1-42 peptides (700254, Invitrogen).

Table S1. Number of animals used in the intracerebral injection studies

Biomaterial	Group	Day	Number of Animals
Fibroin-Ink	Control	1	10
		7	7
		15	10
	MCAO (Stroke)	1	6
		7	6
		15	10
	Control	1	6
		7	5
		15	3
	5xFAD Alzheimer	1	3
		7	2
		15	3
Collagen-Ink	Control	1	8
		15	8
	MCAO (Stroke)	1	5
		15	9
Fibroin-Rho	Control	1	5
		15	5
	MCAO (Stroke)	1	4
		15	4

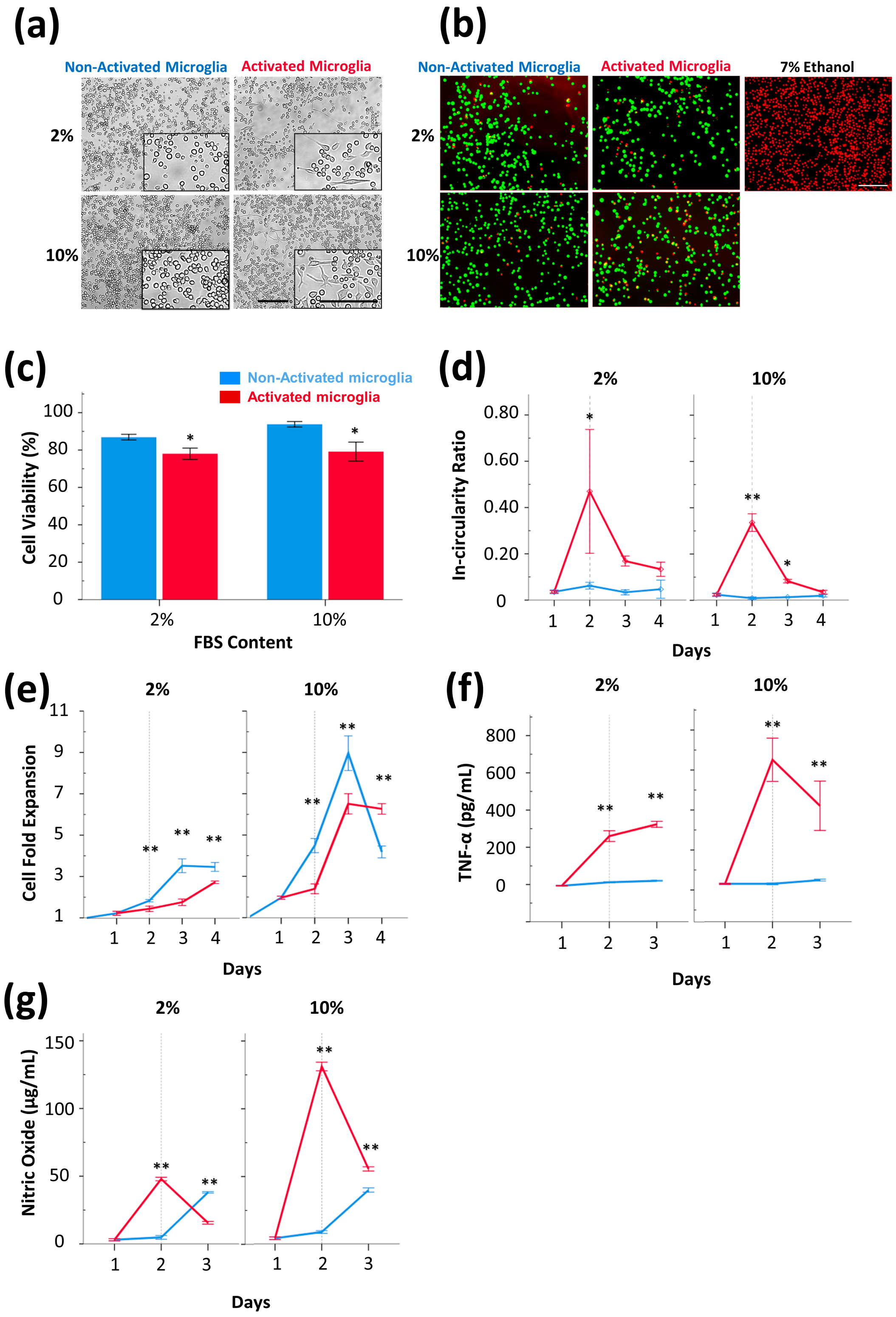
(a)
Non-Activated Microglia
Activated Microglia
2%
10%
(b)
Non-Activated Microglia
Activated Microglia
7% Ethanol
2%
10%
(c)
Non-Activated microglia
Activated microglia
Cell Viability (%)
FBS Content
(d)
2%
10%
In-circularity Ratio
Days
(e)
2%
10%
Cell Fold Expansion
Days
(f)
2%
10%
TNF-α (pg/mL)
Days
(g)
2%
10%
Nitric Oxide (μg/mL)
Days

Figure S1. In vitro characterization of lipopolysaccharide (LPS)-induced microglial activation. (**a**) Representative images at low (scale bar 200 µm) and high (scale bar 100 µm) magnification of BV2 microglia cells non-treated (blue) or treated (red) with LPS incubated with low (2%) and high (10%) serum concentrations. (**b**) Representative fluorescence microscopy images of BV2 cells untreated (non-activated) or treated (activated) with LPS and different FBS concentrations (2% and 10%). Cells were stained with Calcein (survival indicator) and propidium iodide (cell death tracker). The third column shows a positive control of cell mortality after incubation with 7% ethanol. Scale bar: 100 µm. (**c**) Percentage of cell viability 24 hours after LPS treatment (Student´s t-test; asterisks denote significant differences between untreated and treated groups). (**d**) In-circularity ratio, (**e**) cell fold expansion suggestive of BV2 proliferation, (**f**) TNF alpha, and (**g**) Nitric oxide (NO) secretion over time in non-treated and LPS-treated BV2 cells with different serum concentrations. At least six samples per group and temporal point were used in the different studies except for the TNF alpha content, which was determined from 3 samples. Data are shown as the means ± standard error of the mean (SEM). In panels **d–g** (ANOVA with Tukey's post-hoc test), asterisks denote significant differences among different groups with respect to baseline (Day 1: D1); * $p < 0.05$; ** $p < 0.01$).

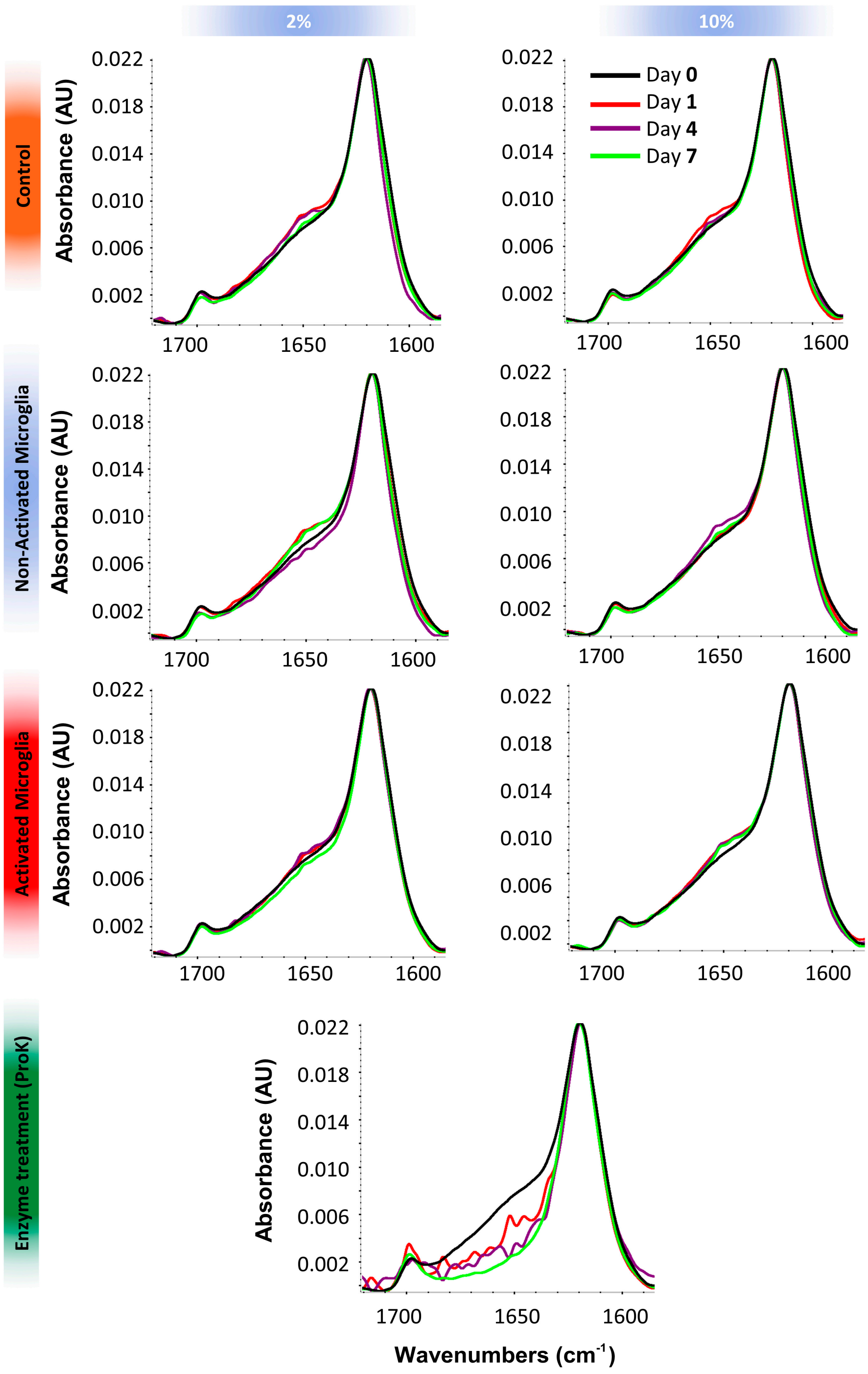

Figure S2. ATR-FTIR spectra of silk fibroin hydrogels across seven days after incubation in control médium, non-activated and activated microglía, and proteinase K. Note that the degradation induced by proteinase K (pro K) was associated with a reduction of absorbance in the regions corresponding of non β-sheet structures (see Figure 1: secondary structure assignments of amide I band components in silk fibroin proteins).

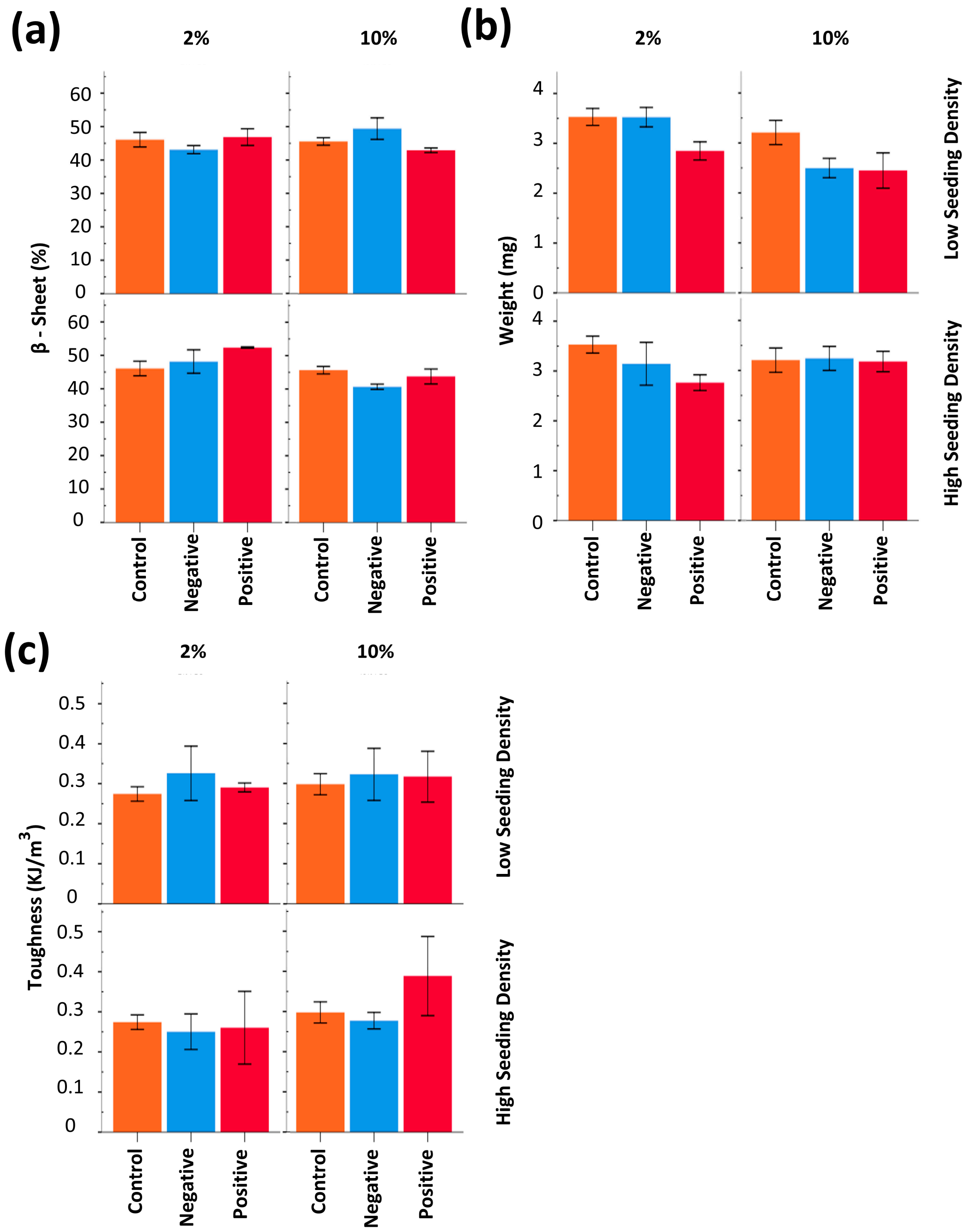

(a)
2%
10%
β - Sheet (%)
0
10
20
30
40
50
60
Control
Negative
Positive
(b)
2%
10%
Weight (mg)
0
1
2
3
4
Low Seeding Density
High Seeding Density
Control
Negative
Positive
(c)
2%
10%
Toughness (KJ/m3)
0
0.1
0.2
0.3
0.4
0.5
Low Seeding Density
High Seeding Density
Control
Negative
Positive

Figure S3. Analysis of silk fibroin hydrogels exposed to conditional medium from high concentration of BV2 microglial cells. (**a**) Percentage of β-sheet, (**b**) weight, and (**c**) toughness of silk fibroin hydrogels seven days after incubation with a conditional medium from $1x10^4$ (low seeding density) and $5x10^4$ (high seeding density) BV2 cells. Blue and red bars represent SF hydrogels treated with non-activated (-LPS) and activated (+LPS) microglia respectively, using different FBS concentrations (2% and 10%). Untreated hydrogels are represented by orange bars. At least seven samples were used per group. Statistical significance was examined via ANOVA.

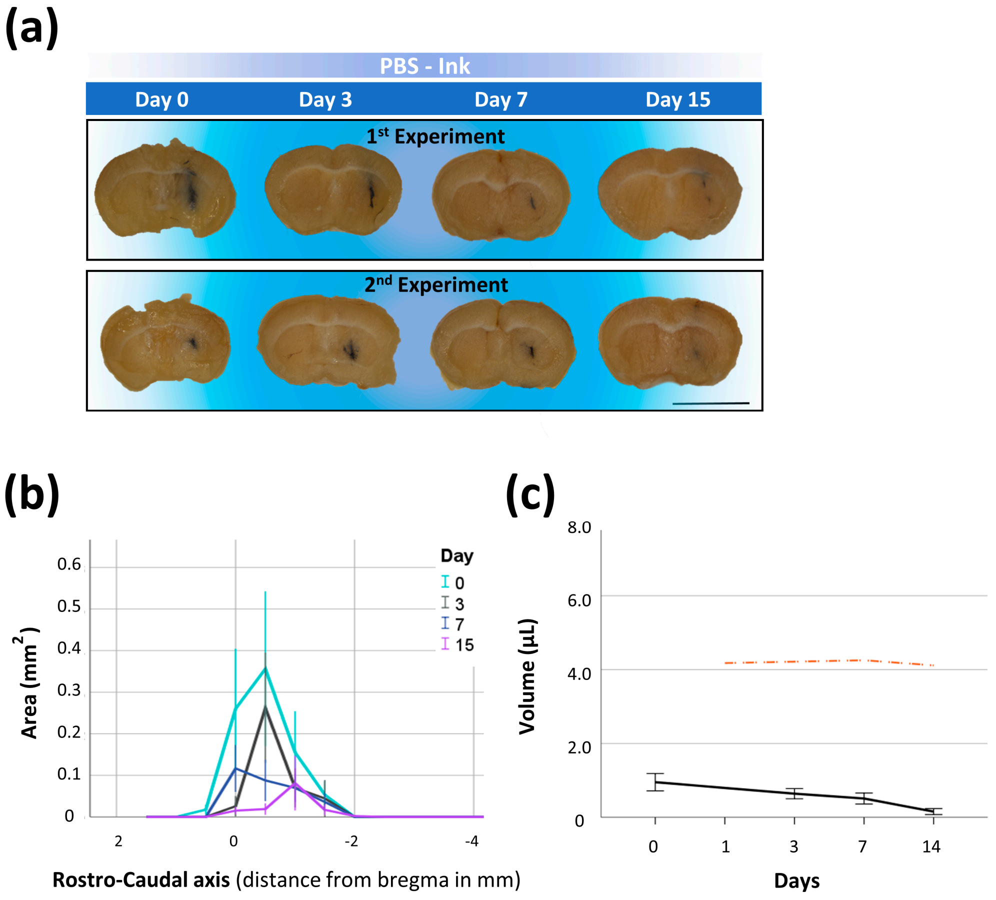

Figure S4. Temporal course of black ink clearance after cerebral injection. (**a**) Representative coronal brain sections of healthy mice at 0, 3, 7, and 15 days after striatal injection of ink alone (scale bar 5mm). (**b**) The plot shows the area occupied by carbon ink across the rostrocaudal axis at different time points after injection (0, 3, 7, and 15 days). On the x-axis, zero indicates the anatomic reference bregma, which is coincident with the point of ink injection. A population of 5-6 injected mice distributed in two independent experiments was used per temporal group. (**c**) Estimation of the carbon ink volume in the brain of healthy mice across time after injection. The orange dashed line indicates the calculated volume (reference value) of intra-striatal ink (reconstituted in silk hydrogels) over time after implantation (data extracted from Figure 6C, Control-Left). Data are shown as the means ± SEM.

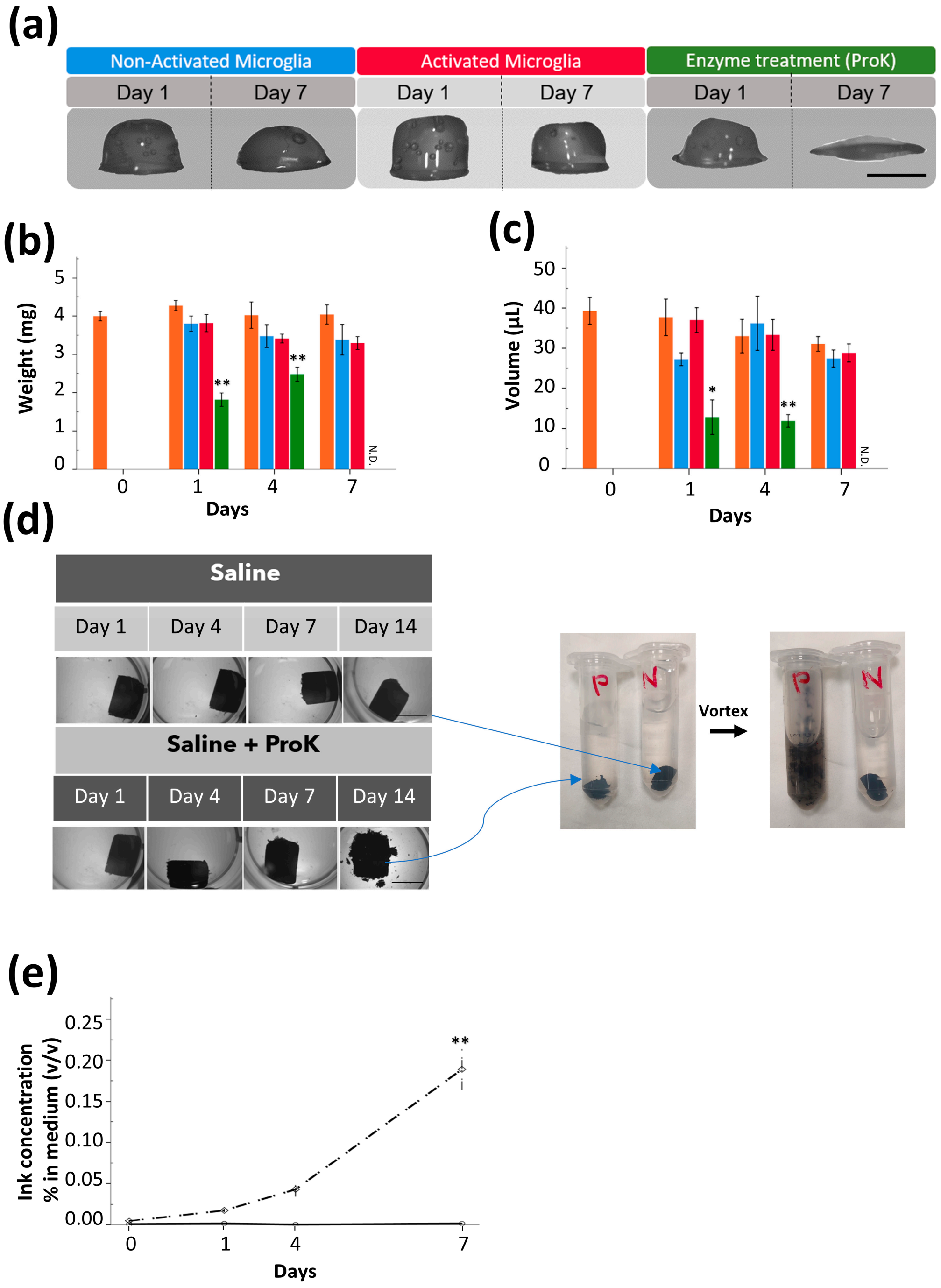
(a)
Non-Activated Microglia
Activated Microglia
Enzyme treatment (ProK)
Day 1
Day 7
(b)
Weight (mg)
Days
N.D.
(c)
Volume (µL)
Days
N.D.
(d)
Saline
Day 1
Day 4
Day 7
Day 14
Saline + ProK
Vortex
P
N
(e)
Ink concentration
% in medium (v/v)
Days

Figure S5. In vitro stability of collagen hydrogels. (**a**) Lateral view of collagen hydrogels showing morphological changes at 1 and 7 days after incubation with a non-activated (blue) and activated-(red) microglia-derived medium, or in the presence of Proteinase K (green) (scale bar 3 mm). Weight (**b**) and volume (**c**) of collagen hydrogels across time after treatment. Blue, red, and green bars represent collagen hydrogels treated with non-activated microglia, activated microglia, and Proteinase K respectively. Untreated hydrogels are represented by orange bars. (**d**) Left, representative top views of collagen hydrogels reconstituted with ink and incubated in PBS (saline solution) or in the presence of proteinase K (ProK) during 14 days of incubation (scale bar 4mm). Right, treatment of collagen hydrogels with Proteinase K (P tube) does not disrupt the whole structure at the macro level, but evidence of degradation is noted upon vortexing the enzyme-treated samples, while the non-treated collagen (N tube) almost remains intact. (**e**) Quantification of the amount of released ink in the solution in the presence and absence of proteinase K. Data are shown as the means ± SEM. Statistical significance was evaluated by a two-way ANOVA with Tukey's post-hoc test (at least five samples were used per group and temporal point in panels **b** and **c**, except for Proteinase K with three samples per temporal point), and five samples per group and temporal point in panel **e**). The asterisks denote significant differences between different groups with respect to untreated collagen (Day 0 in panels **b** and **c**) or with respect to baseline (Day 0 in panel **e**); * $p < 0.05$; ** $p < 0.01$.

References

[1] J.A. Barios, L. Pisarchyk, L. Fernandez-Garcia, L.C. Barrio, M. Ramos, R. Martinez-Murillo, D. Gonzalez-Nieto, Long-term dynamics of somatosensory activity in a stroke model of distal middle cerebral artery oclussion, J Cereb Blood Flow Metab 36(3) (2016) 606-20.
